# Benchmarking for accountability on obesity prevention: evaluation of the Healthy Food Environment Policy Index (Food-EPI) in Australia (2016–2020)

**DOI:** 10.1017/S136898002100447X

**Published:** 2022-02

**Authors:** Janelle Kwon, Erica Reeve, Davina Mann, Boyd Swinburn, Gary Sacks

**Affiliations:** 1 Global Obesity Centre, Institute for Health Transformation, Deakin University, 221 Burwood Highway, Geelong, VIC 3125, Australia; 2 School of Population Health, University of Auckland, Auckland, New Zealand

**Keywords:** Benchmarking, Accountability, Policy process, Obesity prevention, Policy learning, Australia, Healthy food environment

## Abstract

**Objective:**

Despite broad agreement on the need for comprehensive policy action to improve the healthiness of food environments, implementation of recommended policies has been slow and fragmented. Benchmarking is increasingly being used to strengthen accountability for action. However, there have been few evaluations of benchmarking and accountability initiatives to understand their contribution to policy change. This study aimed to evaluate the impact of the Healthy Food Environment Policy Index (Food-EPI) Australia initiative (2016–2020) that assessed Australian governments on their progress in implementing recommended policies for improving food environments.

**Design:**

A convergent mixed methods approach was employed incorporating data from online surveys (conducted in 2017 and 2020) and in-depth semi-structured interviews (conducted in 2020). Data were analysed against a pre-defined logic model.

**Setting:**

Australia.

**Participants:**

Interviews: twenty stakeholders (sixteen government, four non-government). Online surveys: fifty-three non-government stakeholders (52 % response rate) in 2017; thirty-four non-government stakeholders (36 % response rate) in 2020.

**Results:**

The Food-EPI process involved extensive engagement with government officials and the broader public health community across Australia. Food-EPI Australia was found to support policy processes, including as a tool to increase knowledge of good practice, as a process for collaboration and as an authoritative reference to support policy decisions and advocacy strategies.

**Conclusions:**

Key stakeholders involved in the Food-EPI Australia process viewed it as a valuable initiative that should be repeated to maximise its value as an accountability mechanism. The highly collaborative nature of the initiative was seen as a key strength that could inform design of other benchmarking processes.

Obesity and diet-related non-communicable diseases are important preventable risk factors for death and disease globally^(1)^. It is well established that unhealthy food environments are a major driver of escalating rates of diet-related disease^(2)^. A comprehensive policy response will be required to transform the healthiness of food environments, promote healthier diets and reduce diet-related disease^(3,4)^. However, to date, government implementation of recommended policies to improve food environments has been fragmented and insufficient^(3,5,6)^. The limited policy progress reflects the multiple, deeply rooted barriers to obesity prevention policy change. These barriers include food industry lobbying and influence over policy processes, prevailing political ideology that privileges market freedom and minimal government intervention, and frequent framing of diet as an issue of personal choice and responsibility^(7,8)^.

Action by civil society actors (such as researchers and health-related non-government organisations) is recognised as an important contributor to countering barriers to policy change and stimulating government policy commitment^(3,9)^. The International Network for Food and Obesity/NCD Research, Monitoring and Action Support is a collaboration of public health researchers that aims to benchmark the characteristics of food environments internationally as part of efforts to increase recommended policy action^(10)^. In 2013, the International Network for Food and Obesity/NCD Research, Monitoring and Action Support developed the Healthy Food Environment Policy Index (Food-EPI) tool and initiative to assess government policies and actions for creating healthy food environments^(11)^. The Food-EPI tool comprises over forty indicators of good practice policy, which are structured under seven ‘policy domains’ (food composition, food labelling, food promotion, food prices, food provision, food retail, food trade and investment) and six ‘infrastructure support domains’ (leadership, governance, monitoring and intelligence, funding and resources, platforms for interaction and health-in-all-policies). Using the Food-EPI initiative, government progress is assessed against each of the indicators by a panel of experts, and identified gaps are used to develop and promote recommended government policy priorities. As of August 2021, Food-EPI had been applied in over twenty countries^(12,13)^. In Australia, Food-EPI was first implemented in 2016–2017 and included an assessment of the policies of the nine major jurisdictions of Australia including: the Australian Federal Government and each of the eight state and territory governments^(14)^. This was followed up by a 2018–2019 assessment of progress made in each jurisdiction since the first set of reports^(15)^ (refer to online supplementary material, Supplemental File 1 for an overview and summary of the Food-EPI Australia initiative).

Although government policy processes are complex and influenced by many factors, it is important to evaluate strategies designed to support policy change in order to understand what works and identify ways to refine and improve impact^(16)^. To date, globally, there has not been an evaluation of Food-EPI to understand to what extent, and how, it has achieved the intended purpose of contributing to policy change. The aim of this study was to evaluate the impact of the Food-EPI Australia initiative (over the period 2016–2020), including engagement with the initiative, the extent to which the initiative contributed to policy change and ways in which the initiative could be strengthened to increase utilisation and impact.

## Methods

### Study design overview

**Fig. 1:**
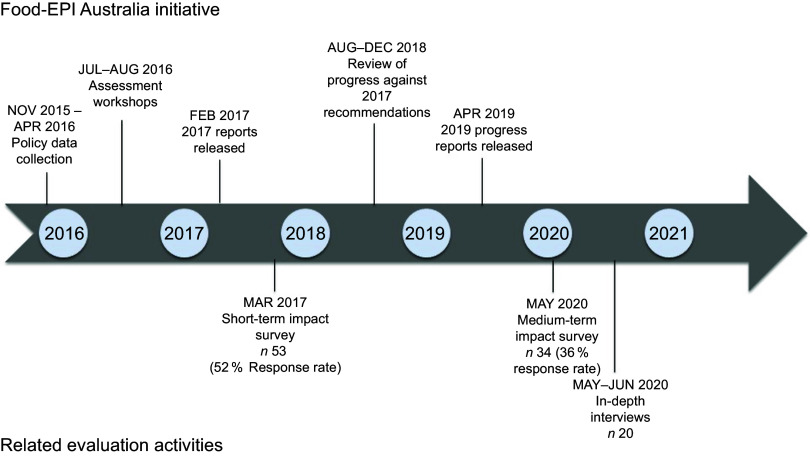
Timeline of Healthy Food Environment Policy Index (Food-EPI) Australia initiative and evaluation activities

This study employed a convergent mixed method design to enable insight into the short- and medium-term impact (1 to 3 years) of the initiative^(17)^. Data sources included online surveys and semi-structured in-depth interviews with government and non-government stakeholders that had been involved in the Food-EPI Australia initiative (described in detail below). These were supplemented with data regarding the level of engagement with the initiative, including the extent of media engagement and correspondence with participating stakeholders (refer to online supplementary material, Supplemental File 1). An overview of the sequence of data collection is provided in Fig. 1.

### Theoretical approach

**Fig. 2:**
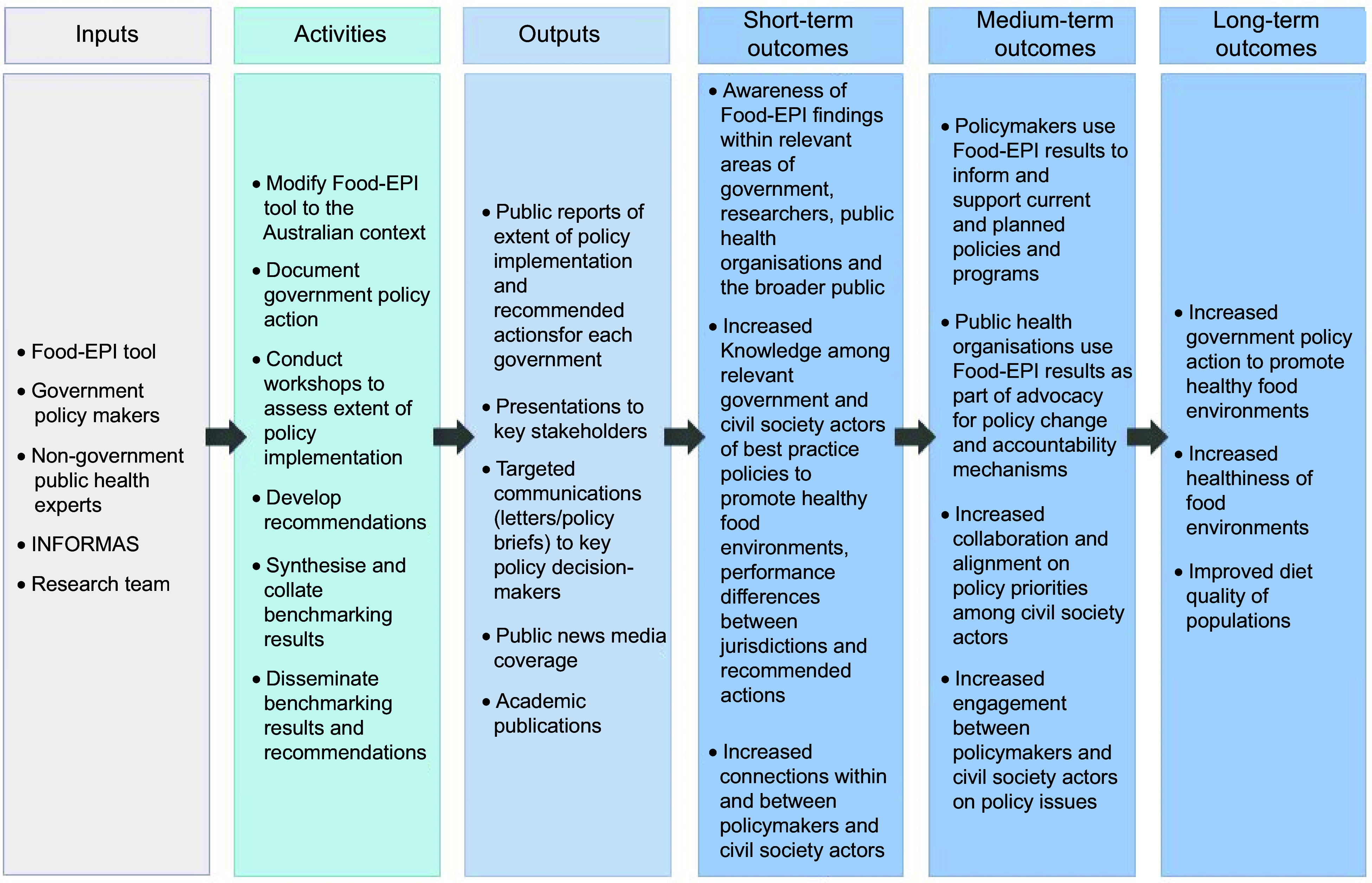
Overview of logic model for Healthy Food Environment Policy Index (Food-EPI) Australia initiative, indicating inputs, activities, outputs and expected outcomes

This study utilised a theory-driven evaluation approach (also known as programme-theory evaluation) to elucidate the extent to which the Food-EPI Australia initiative had an effect in bringing about intended change^(18)^. This theoretical approach was used to develop a logic model for the Food-EPI Australia initiative (Fig. 2), based on the logic model for the International Network for Food and Obesity/NCD Research, Monitoring and Action Support ^(10,12)^. The logic model was used to plan the evaluation and as an initial basis for analysing the data collected.

### Data sources

#### Surveys

Online surveys of non-government stakeholders, including a combination of open-ended and multiple-choice questions (see online supplementary material, Supplemental File 2), were conducted at two time points (2017 and 2020). All non-government stakeholders (*n* 101, refer to online supplementary material, Supplemental File 1 for further details) that contributed to the assessment of government policies and actions were invited to complete both surveys. In March 2017, fifty-three non-government stakeholders (52 % response rate) completed the first survey that sought feedback on the Food-EPI Australia initiative and explored the impact of the initiative on knowledge and professional relationships in the short term (<6 months since first engagement with the initiative). In April–May 2020, thirty-four non-government stakeholders (36 % response rate) completed the second survey that explored the impact and utilisation of Food-EPI Australia over the medium term (up to ∼3 years since first engagement with the initiative). Respondents were also asked to identify opportunities to strengthen the initiative as part of this survey.

#### Interviews

In May–June 2020, semi-structured in-depth interviews were conducted with a purposive sample of government and non-government stakeholders that had been involved in the Food-EPI Australia initiative. Government officials acting as primary representative for each jurisdiction involved in the initiative were invited to participate, and four requested participation of additional government stakeholders that had also been involved. As a result, four of the interviews were conducted in small groups of two–four participants. In total, sixteen government stakeholders participated across nine separate interviews. All government participants were health department officials with at least one participant representing each state, territory and the federal government in Australia.

Non-government stakeholders (*n* 4) were selected for participation in the interviews based on their involvement in assessing government policy action as part of Food-EPI Australia or their extensive expertise working in the area of obesity prevention policy. All non-government interview participants were senior employees at health-related non-government organisations in Australia. Interview discussions were guided by a semi-structured interview guide (online supplementary material, Supplemental File 3), designed to assess outcomes outlined in the logic model. The duration of interviews ranged from 21 to 63 min. The interviews were conducted through video call and were audio recorded and transcribed verbatim.

### Data analysis and synthesis

We applied a hybrid deductive–inductive thematic analysis approach^(19)^ to analyse the data using data management software, NVivo 12. Data were integrated by reviewing all data in accordance with each of the short- and medium-term outcomes of the logic model (Fig. 2). We coded data against the *a priori* logic model, with new codes added inductively as required. We then analysed the data with respect to our objectives and synthesised the themes that emerged as most indicative of the short- and medium-term outcomes. The results are presented against each of the themes, including: (1) engagement with the Food-EPI Australia initiative; (2) awareness of the Food-EPI Australia results; (3) Food-EPI Australia as a knowledge tool; (4) Food-EPI Australia as a tool for facilitating collaboration and consensus; (5) Food-EPI Australia as a tool for informing policy processes; (6) Food-EPI Australia as an advocacy and accountability tool; (7) contribution of Food-EPI Australia to policy change and (8) opportunities to strengthen Food-EPI Australia.

## Results

### Engagement with the initiative

All nine eligible governments nominated a representative to support the Food-EPI Australia initiative. Nominated representatives were predominantly from the chronic disease prevention area within the health department of each jurisdiction. We found that these representatives played a key role in the Food-EPI Australia assessment processes by providing evidence of government policies and actions within the health department and across other areas of government and by brokering wider engagement across government in relation to the initiative. These representatives also acted to secure senior decision-maker agreement to support the initiative by highlighting its potential benefits and addressing any concerns that were expressed by government officials during the implementation of the initiative.

There were also high levels of engagement with the Food-EPI Australia initiative among non-government stakeholders. In 2016–2017, 101 public health experts from fifty-three organisations contributed to the assessment of government policies across eight assessment workshops (refer to online supplementary material, Supplemental File 1 for further details). Government participants indicated that the early commitment of some jurisdictions to be involved in the Food-EPI Australia initiative helped to facilitate the participation of other jurisdictions. Interview respondents indicated that the willingness of participants to be involved in the Food-EPI Australia initiative was further facilitated by its affiliation with the International Network for Food and Obesity/NCD Research, Monitoring and Action Support and by the availability of reports from the successful completion of Food-EPI in New Zealand^(6)^ that provided a clear understanding of the likely outputs of the initiative.

### Awareness of the results of the initiative

The public release of the Food-EPI Australia reports received considerable media attention across Australia (refer to online supplementary material, Supplemental File 1 for further details). This ensured widespread awareness of the initiative among the public health community and the public in general. However, state government interview participants felt that the majority of media coverage had focused on federal policies and, thus, the media coverage itself did not garner substantial ministerial-level attention at the state/territory government-level.

According to government interviewees, there was broad awareness of the Food-EPI Australia reports among government policymakers and officials. Awareness among senior decision-makers was corroborated through the multiple formal letters (from six jurisdictions) received by the project team both after the assessment workshops and in response to the reports sent to government ministers. According to interviewees, many senior decision-makers became aware of the reports because of briefings prepared in response to the public launch of the results. Participants also indicated that briefing processes helped to increase visibility of the initiative at other levels within the bureaucracy due to administrative processes that required briefings to be reviewed and approved at several levels of authority prior to the final senior decision-maker. The delivery of the Food-EPI Australia reports directly to government ministers was also identified as having helped increase awareness of the Food-EPI Australia results among multiple government stakeholders, particularly because officials of increasing authority were required to review and approve responses to ministerial correspondence.

‘[When these sorts of reports are sent to the Minister] you’re forced to do a ministerial briefing on it. That’s good because it means that all the people up through that line see it and are more likely to engage with it’. (Government stakeholder 1, Jurisdiction 1)

Government interview participants indicated that outside of the formal processes in relation to briefings and ministerial correspondence, they received very little interest in the results of Food-EPI Australia from senior decision-makers beyond the public or preventative health areas. Several government interviewees suggested that this may have reflected the challenges of gaining political engagement on obesity prevention issues more broadly, rather than specific limitations of Food-EPI Australia. It was suggested by government interview participants that the 2019 progress update reports may have received less attention than the 2017 reports, having become less novel over time.

### Tool for increasing knowledge

Our findings suggested that Food-EPI Australia was widely utilised by both government and non-government stakeholders as a knowledge product and to facilitate policy learning. Government interviewees noted that, as Food-EPI Australia brought a wide range of policy information together in one place, it was helpful for educating policymakers in nutrition policy, particularly demonstrating the complexity and breadth of policy areas related to food environments. Additionally, the simple scorecard approach was noted as useful to communicate the information in a succinct and clear manner, which was identified as particularly important for senior decision-makers. Food-EPI Australia was also identified to have provided new insights for officials in the health department in relation to policies typically led outside of the health sector, including nutrition education in schools, land-use planning and political donations.

‘I had a pretty good understanding in healthcare facilities; that’s been my main area that I’ve been working in, so healthcare facilities, policies, healthy eating policies. [I’ve worked in relation to] schools to a lesser extent, so there were some interesting [lessons] within the Food-EPI report around schools’. (Government stakeholder 7, Jurisdiction 4)

Survey results suggested that Food-EPI Australia had also been useful in increasing policy knowledge among non-government stakeholders. In 2017, the majority of survey respondents ‘agreed’ or ‘strongly agreed’ that Food-EPI Australia had increased their knowledge of food environments and related policy in their state or territory (76 %), at the federal/national level (81 %) and of current best practice (83 %) (online supplementary material, Supplemental File 4, Table 1 and Fig. 1). In 2020, the majority of survey participants ‘agreed’ or ‘strongly agreed’ that Food-EPI Australia had increased their understanding of policy action among Australia governments (85 %) and of best practice policy to promote healthy food environments (85 %), although a small number of survey participants (6 %) disagreed (online supplementary material, Supplemental File 4, Table 2 and Fig. 2). Additionally, several non-government interviewees noted that Food-EPI Australia provided them with important insight into jurisdictional progress, helping them to develop stronger advocacy campaigns.

Food-EPI Australia was also identified to have supported knowledge development among individuals not directly involved in nutrition policy, with several non-government interview participants indicating that they had used Food-EPI Australia in education settings, such as higher education teaching and professional development training for healthcare workers.

### Tool for increasing collaboration and consensus

Interviewees and survey respondents reported that the Food-EPI Australia processes had facilitated collaboration across stakeholders. For instance, the assessment workshops, which brought together multiple stakeholders to discuss a topic of shared interest, reportedly helped to strengthen and facilitate new professional connections among non-government stakeholders. In 2017, a substantial proportion of survey respondents agreed that they had made new connections or strengthened existing connections with non-government professionals and people working for government (43 % in 2017 and 38 % in 2020) (online supplementary material, Supplemental File 4, Table 1 and Fig. 1). Additionally, several government interviewees reported that the process of collecting policy evidence from across government helped to establish new cross-sectoral relationships with officials outside of their department. These relationships were identified by participants as critical for responding to relevant policy issues, or when planning whole-of-government initiatives.

In 2020, survey participants also indicated that Food-EPI Australia had helped to increase collaboration between government and non-government stakeholders (85 %) and promoted alignment of advocacy efforts among non-government organisations (73 %), with only a small proportion (<10 %) of participants indicating their disagreement (online supplementary material, Supplemental File 4, Table 2 and Fig. 2). Interviews with key stakeholders revealed that Food-EPI Australia facilitated policy alignment through informal and unstructured ways. For example, interview participants reported cross-checking planned policies or advocacy campaigns with Food-EPI Australia to ensure alignment. Additionally, several government participants described using Food-EPI Australia to inform discussions regarding the development of a national obesity strategy.

### Tool for informing policy processes

Interview participants reported that Food-EPI Australia was one of many sources of information being used to inform evidence-based policy approaches.

‘Policy is informed by a range of different things including stakeholder views and a whole stack of evidence, and this [Food-EPI Australia] is one of the evidence tools, which was very useful because it was a practical evidence tool, used to inform policy’. (Government stakeholder 12, Jurisdiction 6)

Government interview participants reported that they had used Food-EPI Australia as a ‘blueprint’ for planning work or strategies, to take stock of government progress and identify gaps and to cross-check policies under development for consistency with the recommended policies and gaps identified in the Food-EPI reports. Some government interviewees reported using the good practice policy examples from other jurisdictions and countries to generate ideas for how policy could be formulated or implemented in their jurisdiction.

Several government interviewees also described using Food-EPI Australia as a reference in policy documents to substantiate the merits of a particular policy decision or to substantiate policy directions that had already been decided. The independence of the report was highlighted as particularly important for providing authoritative evidence to validate policy decisions.

‘It legitimises the pursuit of particular policies because…this tool [Food-EPI Australia] has been developed based on evidence and research and these are the domains for action. They’re not things that someone’s just dreamt up’. (Government stakeholder 15, Jurisdiction 8)

Interviews revealed differing levels of utilisation of Food-EPI Australia across jurisdictions. Some government informants, particularly those from jurisdictions where the political party in power had changed since the first Food-EPI Australia assessment, described using Food-EPI Australia as part of efforts to progress new policies. In contrast, informants from another jurisdiction felt that the current political environment did not provide an opportunity to leverage the Food-EPI Australia data and indicated that they used the reports to gain recognition for and continue existing work, rather than to progress new policy.

### Tool for advocacy and accountability

In 2020, the majority (65 %) of survey participants reported using Food-EPI Australia as part of direct advocacy to government decision-makers (online supplementary material, Supplemental File 4, Table 2 and Fig. 3). Examples of policies being implemented by governments within the Food-EPI Australia reports were identified as particularly helpful for supporting advocacy efforts by stakeholders outside of government, especially when they could be compared to a lack of action by another government.

‘It was useful to have examples to promote in media interviews and also to send to politicians who were somewhat reluctant to take action’. (Non-government survey participant 3)

‘I think being able to compare state jurisdictions was definitely the most important part [of Food-EPI Australia]. And that way of being able to show the stronger states and the weaker states was its most important contribution’. (Non-government interview participant 2)

Government interview participants recognised the importance of Food-EPI Australia for maintaining ‘noise’ and political attention on policies for healthy food environments, and one government interview participant described using it to highlight accountability for taking action.

‘[There have been] a few occasions where, as part of the rationale for progressing something forward, I have referred to Food-EPI, not as the only thing, but just as one of the things. [I’ve said] “Oh, and we are being monitored on this, and scorecarded.” So yes, I guess it’s been an extra incentive [for taking action]’. (Government stakeholder 8, Jurisdiction 5)

However, several government interviewees suggested that the political accountability associated with Food-EPI Australia was expected to increase over time, as the rating exercise is repeated.

### Contribution to policy change

While it was not possible to directly attribute policy change to Food-EPI Australia, interviews with government policymakers provided a number of examples where Food-EPI Australia contributed to significant shifts in policy. For instance, one policymaker used the Food-EPI Australia information to justify the need for additional public health nutrition resources.

‘I drew [a senior decision-makers] attention to [the Food-EPI Australia results] when I was advocating to get more public health nutrition positions within our branch’. (Government stakeholder 10, Jurisdiction 5)

Another policymaker described using the Food-EPI Australia recommendations to suggest nutrition policy priorities as part of a state-based grants programme. The relevant priorities of the programme that were ultimately announced aligned with the policy recommendations of Food-EPI Australia.

‘I would say that the fact that we funded a [policy] along the lines of what [Food-EPI Australia] had recommended might have been because of that piece of advice’. (Government stakeholder 12, Jurisdiction 6)

In addition, examples were given of where Food-EPI Australia had proved valuable in supporting the continuation of policy. Several interviewed policymakers described using the recognition of the merit of current policies by Food-EPI Australia to highlight the importance of those policies and justify their continued funding.

‘[Food-EPI Australia]’s really helped keep [an existing policy] alive at a time when there was quite a bit of pressure to disinvest…. in briefings we did highlight that the [existing policy at risk of defunding] and [another existing policy] were the two areas that we scored well in, and yeah, we communicated that up to the [senior policymaker]’. (Government stakeholder 11, Jurisdiction 5)

While non-government stakeholders reported using Food-EPI Australia to highlight jurisdictional differences in policy implementation, and advocate for related policy change, it is not clear how influential this strategy was. Although some government informants noted strong interest in jurisdictional differences among senior decision-makers, others suggested there was minimal interest in such comparisons. Some government interview participants also raised concerns with the appropriateness of jurisdictional comparisons due to varying characteristics and challenges between jurisdictions (e.g. geographic or population size).

Importantly, government interview participants identified several salient factors unrelated to Food-EPI Australia that either reduced the propensity for food environment policy change (e.g. government values that prioritise economic prosperity and health service delivery), or conversely, increased opportunities to progress policy (e.g. change in governing political party).

### Opportunities to strengthen the initiative

Interview and survey participants were almost universally supportive of the continuation of Food-EPI Australia through repeated assessments. Several opportunities were offered to strengthen the rigour and impact of the initiative in the future. Participants suggested changes to the arrangement and composition of the assessment panels to improve reliability of assessments, including that assessment panels include representation across all expert knowledge areas relevant to Food-EPI Australia. Participants also recommended that Food-EPI Australia aims for greater consistency in the process across jurisdictions. For example, government interviewees noted potential for substantial variation between assessment panels across jurisdictions with regard to their policy knowledge and/or working relationships. Other government participants raised practical challenges related to the variation in jurisdictional characteristics and policy challenges, and the difficulty in assessing policies that had joint responsibility between federal and state/territory governments. They also felt there was scope to improve the criteria by which government policy progress was scored. Several interview participants felt that the assessment focused heavily on whether policies were in place without enough consideration of the content or effectiveness of those policies. They felt that there was not sufficient time in the assessment workshops to discuss such complexities or thoroughly interrogate particular policy issues due to the breadth of policy topics covered.

Participants suggested that the dissemination of the reports and findings could have been strengthened. Over 60 % of 2020 survey respondents suggested the initiative could be improved by a greater focus on dissemination and knowledge translation (online supplementary material, Supplemental File 4, Fig. 4). Suggestions for strengthening dissemination centred around increasing media impact through a more strategic and targeted media release tailored to each state and/or focused on single policy issues, and more frequent and ongoing communication between the Food-EPI Australia project team and public health professionals.

## Discussion

This evaluation of the impact of the Food-EPI Australia initiative (2016–2020) demonstrated a number of ways in which the initiative had been useful in informing and guiding obesity prevention policy in Australia. We found that policymakers across multiple jurisdictions had used the Food-EPI Australia findings in varying degrees to inform policy development processes, identify policy gaps and support policy decisions. We also found that civil society actors used the evidence of government progress and examples of implemented good practice policies to strengthen advocacy efforts. Overall, Food-EPI Australia has helped to increase knowledge, facilitate policy learning and encourage policy coherence among stakeholders. These findings suggest Food-EPI Australia is a valuable initiative that can contribute to implementation of globally recommended policies for obesity prevention.

While Food-EPI Australia was found to contribute to several mechanisms that could support policy change, the impact on policy learning emerged as a particularly important contribution. Specifically, Food-EPI Australia helped to increase knowledge of good practice policy to promote healthy food environments among policy actors engaged in obesity prevention policy processes. These policy actors, in turn, utilised the information to broker wider policy learning among senior decision-makers. The importance of policy learning is consistent with assertions from the political science literature that policy-oriented learning, whereby beliefs about policy are altered in response to new information or experience, is a key pathway to policy change^(20)^. According to the Advocacy Coalition Framework^(21)^, a widely applied political science framework to understand and explain policy change in relation to highly complex and contested policy issues^(22)^, policy-oriented learning most often alters beliefs about secondary aspects of policy, such as the particular policy design or instrument, rather than fundamental belief systems (that are more resistant to change)^(20)^. Consequently, and as observed in this study, policy-oriented learning can only be expected to bring about relatively minor changes in policy^(23)^.

This study identified that the Food-EPI Australia initiative successfully brought together a large number of public health policy advocates as part of formal assessment processes, and that the Food-EPI reports provided shared resources for advocacy. While stakeholders predominantly reported using the Food-EPI Australia findings independently to inform policy and advocacy strategies, the Food-EPI Australia initiative also helped pave the way for consensus on action needed for obesity prevention, particularly at the national level. For example, in 2017, a number of public health advocates and organisations were undertaking a process to generate consensus on priority obesity prevention policy actions at the national level. This culminated in the release of the Tipping the Scales report in September 2017, which was endorsed by thirty-four health organisations in Australia^(24)^. The policy priorities outlined in the Tipping the Scales report largely reflected the priority recommendations in the 2017 Food-EPI Australia report for the federal government, including a focus on restricting the exposure of children to the marketing of unhealthy foods, and increasing the price of unhealthy foods. The importance of advocacy coalitions is a central tenet of the Advocacy Coalition Framework, which posits that, for policy actors to maximise influence on policy decisions, they need to seek allies to share resources and undertake coordinated action^(20)^. When viewed through the lens of the Advocacy Coalition Framework, the process of implementing the Food-EPI initiative can be seen as a mechanism for building and strengthening an advocacy coalition through bringing together public health experts and policymakers to boost attention to obesity prevention policy options.

This evaluation identified that there is broad support for the continuation of Food-EPI Australia, with key stakeholders cognisant of the initiative’s contribution to evidence and its importance for maintaining political attention on policies for healthy food environments. Nevertheless, there are opportunities to strengthen the initiative in order to increase utility and impact. First, assessment processes should be revised to increase comparability of assessments across jurisdictions in Australia and to ensure that there is appropriate expertise on the panel across the range of policy areas included in the Food-EPI tool. This could be achieved by surveying panel members’ expertise to guide panel selection and ensure an appropriate mix of knowledge. Additionally, establishing a central assessment panel, rather than jurisdiction-based assessment panels, may help to increase comparability of results across jurisdictions in Australia. Online assessments have been utilised in Food-EPI studies in other countries^(6)^ and could help to coordinate diverse expertise across the country for future assessments. Second, dissemination strategies should focus more at the jurisdiction level to optimise political interest. Strategic framing of policy problems and solutions to decision-maker beliefs and broader government priorities can increase acceptability and commitment to policy action^(25–29)^. Targeted media strategies for each jurisdiction should therefore be considered in order to increase relevancy of Food-EPI Australia across a broader range of decision-makers. Third, there was evidence that the Food-EPI Australia initiative lost some novelty among decision-makers after the first assessment. Thus, opportunities to maintain interest and reframe the problem should be further explored^(30)^, including through the incorporation of elements of environmental sustainability. However, thorough consideration of how to implement such changes would be required to ensure the core focus of healthy food environments is not lost.

### Policy implications

As one of the first formal evaluations of accountability initiatives in the area of public health^(12,31)^, this study provides important insights for civil society actors interested in mechanisms to generate increased commitment to public health action. The study findings indicated that the collaborative nature of the initiative and the involvement of a broad range of stakeholders was seen as a key strength that increased the impact of the initiative. Importantly, this study demonstrated that policy benchmarking can contribute to policy change. This is consistent with findings from an evaluation of a benchmarking initiative of food industry policies in Australia, which found that the initiative helped to improve food company nutrition policies^(12,32)^. Together, these studies suggest that policy benchmarking is a useful strategy as part of efforts to advance public health policy and warrants further use and exploration by public health advocates.

Previous studies of obesity prevention policy processes in the Australian context have shown that they are complex and influenced by numerous factors^(26,28,29,33,34)^. Moreover, several Australian studies have identified that there are multiple and substantial barriers to policy change in this area, which has led to the low level of implementation of globally recommended policies^(35–37)^. While this study has shown that the Food-EPI initiative shows promise as a mechanism for influencing change, future studies are needed to gain an in-depth understanding of the role that the Food-EPI initiative plays as part of processes for the development of individual policies, and over the longer term (greater than 3 years). These studies can also further elucidate strategies to amplify the impact of promising initiatives.

### Strengths and weaknesses

The strengths of this study include the comprehensive representation of participants to inform the evaluation, including government policymakers from all state, territory and federal governments, as well as multiple non-government stakeholders. The extended evaluation period, involving multiple data sources, was a further strength that enabled insight into both the short- and medium-term impacts of the initiative.

The evaluation was, however, not without limitations. The first set of limitations relates to the participants. Participants were recruited through their previous involvement in the Food-EPI Australia initiative. While this approach ensured participants could provide detailed comments about the initiative, it also restricted opportunity to understand the impact on stakeholders that were not directly involved in the initiative. Key informant interviews were conducted 3 years after the first assessment, which may have limited how much detailed information or examples participants could recall. However, incorporation of survey data collected shortly after the reports were released in 2017 helped to complement and corroborate findings. The relatively low-response rate to the surveys of non-government stakeholders (52 % response rate in 2017, 36 % response rate in 2020) may also have introduced bias into the results. The second limitation relates to the data sources used to measure the outcomes evaluated as part of the study. We relied on subjective perspectives (through in-depth interviews and surveys) on outcomes such as collaboration and engagement among stakeholders, and the use of Food-EPI Australia findings in policy processes. The rich and nuanced data from the in-depth interviews provided deep contextual information that was valuable in assessing the outcomes of interest, and the surveys provided an efficient mechanism to gather data from a broad range of stakeholders involved in the Food-EPI Australia process. Future studies could also assess these outcomes through more objective means, such as by analysing: the number of documented formal partnerships between stakeholders; the extent to which the Food-EPI findings and recommendations are referenced as part of official policy documents and the advocacy materials of public health organisations. A third limitation of this study is that we did not evaluate the costs or economic credentials of the initiative. Due to the extensive scope and scale of the initiative, many participants contributed substantial time to support the assessments, particularly government representatives that collected and verified a vast array of policy evidence. Efforts should be directed at quantifying the costs and benefits of the initiative so that future evaluations may also assess cost-effectiveness. Finally, transferability of the findings to Food-EPI initiatives in other countries may be limited due to contextual factors related to the Australian political landscape. However, the theoretical grounding of the evaluation is expected to have helped identify lessons that are generalisable to wider policy contexts.

## Conclusion

The findings from this first evaluation of Food-EPI Australia suggest that it was perceived by relevant stakeholders as a valuable initiative that was utilised in multiple ways to inform and guide policy. The initiative increased knowledge of best practice policy and government policy progress directly informed policy processes and acted as a tool to increase decision-maker awareness and support for policy solutions. The highly collaborative nature of the initiative was seen as a key strength that could inform the design of other benchmarking processes.

While public health advocates frequently used evidence from Food-EPI Australia to strengthen advocacy efforts and hold governments accountable, the value of the initiative as an accountability mechanism is likely to be maximised with repeated implementation over time. Future iterations of Food-EPI Australia should seek to modify assessment processes to increase comparability of findings across jurisdictions, reframe media dissemination to more greatly resonate with decision-maker priorities and explore further opportunities for public health advocates to coordinate coherent advocacy messages and activities.

Ultimately, the success of public health advocacy efforts for obesity prevention needs to be measured by the extent to which they lead to meaningful policy change and positive health outcomes. Food-EPI Australia shows promise in contributing to change, and there is likely to be value in ongoing support for the initiative, and in understanding ways to amplify its impact.

## Supplementary material

For supplementary material accompanying this paper visit https://doi.org/10.1017/S136898002100447X. click here to view supplementary material
