# Supplementary material for: Benchmarking for accountability on obesity prevention: evaluation of the Healthy Food Environment Policy Index (Food-EPI) in Australia (2016–2020)
Source: Public Health Nutr. 2021 Oct 28;25(2):488–97. doi: 10.1017/S136898002100447X (PMC8883784; doi:10.1017/S136898002100447X)
Supplement: Supplementary file 1 [file S136898002100447Xsup.zip › S136898002100447Xsup001.docx]

**Supplementary File 3 – Food-EPI Australia evaluation interview schedule**

**Introduction**

Thank you for taking the time to speak with me today. The interview is part of an evaluation of the Food Policy Index project. The evaluation is seeking to understand if and how the project influenced policies and action for obesity prevention, and identify opportunities for improvement for future iterations of the project. Throughout the interview I will use the term Food-EPI when referring to the Food Policy Index project. In terms of scope, when considering the questions, I would like you to consider the entire Food-EPI project including the process of conducting the assessments, the report itself and the dissemination of the results. The interview should take around 30-45 minutes, and you can stop the interview at any time. If you consent, the audio from this interview will be recorded and quotes from this interview may be used when reporting the results of the evaluation. None of the data reported from the interview will be identifiable to you or your organisation. Any questions before I start the recording?

**Questions**

1. To get started, can you tell me a bit about your role and the area of government/organisation that you work in?
2. How have you been involved in Food-EPI? (Differentiate between roles in 2017 and 2019)?
3. From your experience, to what extent was the Food-EPI project noticed within your immediate work team or branch? What about across broader areas of your department/government/public health community?
4. How was interest shown? Can you give me examples of conversations or discussions that demonstrated interest in Food-EPI?
5. Were there any particular aspects of Food-EPI that gained more attention?
6. How have you used Food-EPI in your day-to-day work? What about by others elsewhere in your government/organisation?
7. Do you think Food-EPI had an impact on your knowledge and/or beliefs about what is best practice when it comes to policies to promote healthy food environments? What about the level of policy action or inaction among Australian governments?
8. Thinking about others in your government/organisation, do you think Food-EPI had an impact on knowledge and/or beliefs about what is best practice when it comes to policies to promote healthy food environments? What about the level of policy action or inaction among Australian governments?
9. Do you think Food-EPI had an impact on the extent of collaboration or degree of coherence within your government (among departments or divisions)? What about between governments (i.e. among your counterparts in other states)? (for government participants)

What about between non-government advocates and organisations groups working in this area? (for non-government participants)

1. Can you reflect on what resources you had to contribute to the assessments of government policies and actions conducted as part of Food-EPI, and resources to respond to the results?
2. Thinking back to when the Food-EPI report was released, can you think of any policy issues or external events that may have reduced or increased the relative importance of Food-EPI?
3. How do you think Food-EPI has contributed to policy development or priorities, if at all? Has this changed over time?
4. How could the Food-EPI assessment and report be changed next time to better support policy action?
5. We’re considering adding aspects of environmental sustainability into the existing set of indicators and scoring. What is your view on the importance of that and how it might be received by governments? What about among senior government policy makers?
6. We’re also considering adapting it for use specifically for local governments. How could we ensure it is useful for local governments?
7. Lastly, is there anything else you would like to mention related to Food-EPI that we haven’t discussed yet today?
