# Supplementary material for: Benchmarking for accountability on obesity prevention: evaluation of the Healthy Food Environment Policy Index (Food-EPI) in Australia (2016–2020)
Source: Public Health Nutr. 2021 Oct 28;25(2):488–97. doi: 10.1017/S136898002100447X (PMC8883784; doi:10.1017/S136898002100447X)
Supplement: Supplementary file 1 [file S136898002100447Xsup.zip › S136898002100447Xsup002.docx]

# Supplementary file 1: Overview and timeline of the Food-EPI Australia initiative

The Food-EPI Australia initiative was conducted by researchers at Deakin University, in partnership with the Obesity Policy Coalition (OPC), with funding provided by The Australian Prevention Partnership Centre (TAPPC). The Food-EPI tool and process was adapted for the Australian context and applied in 2016/2017 to assess Australian federal, state, and territory government implementation of policies and actions to promote healthy food environments. The Food-EPI assessment process is described in detail elsewhere^(1)^.

**Initial assessment in 2016/2017**

The Food-EPI Australia process in 2016/2017 involved the collection of policy details in relation to each indicator for each jurisdiction, in cooperation with government representatives, over a period of six months. Each government provided verification of the accuracy and completeness of the policy details collected for their specific jurisdiction. For each indicator, the extent of implementation of policies (compared to international best practice) was assessed for each jurisdiction by local panels of invited public health experts as part of a series of in-person assessment workshops. A separate expert panel was established for each jurisdiction, with each panel assessing the government in their own jurisdiction as well as the Australian Federal Government. Each expert panel then identified and prioritised key recommendations for each of the governments they assessed.

In 2016/2017, 101 public health experts (out of 144 invited, response rate of 70%) from 53 organisations contributed to the assessment of government policies across eight assessment workshops. The expert panel for each jurisdiction consisted of between eight and 19 non-government public health experts (including academics and senior representatives of prominent health-related non-government organisations) who were selected for participation on the basis of their knowledge of policies and practices related to food environments and/or their leadership position within the public health community. Government representatives attended the assessment workshops as observers to verify or clarify information, as required. The assessment results were used to prepare scorecards for each jurisdiction. The scorecards and recommendations were collated into professionally designed reports for each jurisdiction as well as an overall summary report covering all jurisdictions. Key recommendations included the development and implementation of policies to: restrict the exposure of children to the marketing of unhealthy foods, increase the price of unhealthy foods, and improve the healthiness of foods in settings controlled or managed by governments^(2)^.

The reports were publicly released in February 2017 via a media release that was broadly disseminated (in conjunction with the OPC) and a dedicated website for the initiative^(3)^. The results were subsequently summarised into a findings brief prepared in conjunction with TAPPC^(3)^, and presented at a dedicated webinar hosted by TAPPC^(5)^, scientific conferences, and included as part of academic publications^(6)^.

The 48-hour period following the launch of the 2017 Food-EPI Australia reports received significant media coverage. The Federal Government Health Minister, the Federal Government Shadow Minister for Health and the Australian Food and Grocery Council issued media releases in response to the reports on the day of the launch. One-hundred and ninety-four individual media items across print (n=15), television (n=44), radio (n=71) and online (n=64) were identified in the 48 hours following the release of the 2017 project reports^(7)^.

**Progress update in 2018/2019**

In 2018/2019, the project team conducted a process to assess progress made by each jurisdiction since the 2016/2017 assessment. Over a period of four months, the project team collected details, in close consultation with government representatives in each jurisdiction, of actions taken in relation to the recommendations made in the 2017 reports. Each government provided verification of the accuracy and completeness of the evidence collected. The project team then assessed each government’s progress against each recommendation as either ‘limited action’, ‘some action’ or ‘substantive action’. For each jurisdiction, key areas of progress and recommended areas of focus were identified, in conjunction with government representatives. The results were again collated into professionally designed reports for each jurisdiction as well an overall summary report covering all jurisdictions^(8)^. The reports were publicly released in April 2019 and uploaded to the dedicated project website^(3)^. Documents providing the detailed evidence underpinning the assessments for each jurisdiction were also uploaded to the dedicated website. In relation to the media coverage for the 2019 reports, there were 49 individual media items including print (n=10), television (n=8), radio (n=27) and online (n=4) in the 48 hours following release^(7)^.

**Release of each set of results**

A week prior to the public release of each set of results, an embargoed copy of the reports and media release were shared with government representatives in each jurisdiction as well as professional contacts in a range of organisations related to the initiative. After each launch, the project team sent the head of state (Premier, Prime Minister or Chief Minister, as relevant) and Health Minister of each jurisdiction a copy of the report for their government as well as the overall summary report for Australia. In response, the project team received eight letters in return in relation to the 2017 reports and six letters in relation to the 2019 progress reports.

**References**

1. Swinburn B, Vandevijvere S, Kraak V *et al.* (2013) Monitoring and benchmarking government policies and actions to improve the healthiness of food environments: a proposed Government Healthy Food Environment Policy Index. *Obes Rev* 14, 24-37.
2. Sacks G (2017) Policies for tackling obesity and creating healthier food environments: scorecard and priority recommendations for Australian governments. Melbourne: Deakin University and the Obesity Coalition Australia. <https://www.foodpolicyindex.org.au/> (accessed August 2021)
3. Deakin University (2019) Food Policy Index Australia. <https://www.foodpolicyindex.org.au/> (accessed August 2021)
4. The Australian Prevention Partnership Centre (2017) Findings brief: Roadmap to tackle obesity prevention. <https://preventioncentre.org.au/wp-content/uploads/2017/03/1702_FB_SACKS.pdf> (accessed August 2021)
5. Sacks G. How governments can achieve best practice in obesity prevention policy [webinar]. 21 July 2017.
6. Vandevijvere S, Barquera S, Caceres G *et al.* (2019) An 11-country study to benchmark the implementation of recommended nutrition policies by national governments using the Healthy Food Environment Policy Index, 2015-2018. *Obes Rev* 20, 57-66.
7. Deakin University (2019). In the media - Food Policy Index Australia. <https://www.foodpolicyindex.org.au/>media (accessed August 2021)
8. Sacks G & Robinson E (2019) Policies for tackling obesity and creating healthier food environments: 2019 progress update, Australian governments. Melbourne: Deakin University. <https://www.foodpolicyindex.org.au/> (accessed August 2021)
