# Supplementary material for: Benchmarking for accountability on obesity prevention: evaluation of the Healthy Food Environment Policy Index (Food-EPI) in Australia (2016–2020)
Source: Public Health Nutr. 2021 Oct 28;25(2):488–97. doi: 10.1017/S136898002100447X (PMC8883784; doi:10.1017/S136898002100447X)
Supplement: Supplementary file 1 [file S136898002100447Xsup.zip › S136898002100447Xsup003.docx]

**Supplementary File 4 – Food-EPI Australia evaluation survey results**

Table S1: Results from the 2017 online evaluation survey (n=53)

|  | **Strongly disagree (%)** | **Disagree (%)** | **Neutral (%)** | **Agree (%)** | **Strongly agree (%)** |
| --- | --- | --- | --- | --- | --- |
| I have increased knowledge of food environments and related policy in my state/territory | 0 | 9 | 15 | 62 | 13 |
| I have increased knowledge of food environments and related policy at a federal/national level | 0 | 8 | 11 | 57 | 25 |
| I have increased knowledge of current best practice/what other governments are doing internationally | 0 | 4 | 13 | 49 | 34 |
| I have made new connections/strengthened existing relationships with **non-government** professionals | 2 | 21 | 34 | 43 | 0 |
| I have made new connections/strengthened existing relationships with **government** professionals | 2 | 28 | 32 | 38 | 0 |
| My knowledge of food and nutrition policy increased as a result of the project | 0 | 2 | 8 | 75 | 16 |

Table S2: Results from the 2020 online evaluation survey (n=34)

|  | **Strongly disagree (%)** | **Disagree (%)** | **Neither agree nor disagree (%)** | **Agree (%)** | **Strongly agree (%)** |
| --- | --- | --- | --- | --- | --- |
| The project and its findings increased alignment of advocacy efforts between non-government organisations and groups working to improve the healthiness of food environments | 0 | 9 | 18 | 44 | 29 |
| The project and its findings encouraged collaboration/strengthened relationships between different non-government organisations and groups working to improve the healthiness of food environments | 0 | 6 | 9 | 38 | 47 |
| The project and its findings encouraged collaboration/strengthened relationships between government officials and non-government organisations and groups working to improve the healthiness of food environments | 6 | 0 | 9 | 41 | 44 |
| The project and its findings influenced my understanding of what other countries are doing to promote healthy food environments | 0 | 9 | 18 | 44 | 29 |
| The project and its findings influenced my understanding of the level of policy action among Australian governments to promote healthy food | 0 | 6 | 9 | 38 | 47 |
| The project and its findings influenced my understanding of best practice policies to promote healthy food environments | 6 | 0 | 9 | 41 | 44 |
|  | | | | | |
|  | **Unsure (%)** | **Not used at all (%)** | **Used to a small extent (%)** | **Used to a moderate extent (%)** | **Used a lot (%)** |
| Increasing awareness and understanding of best practice obesity prevention policy | 12 | 15 | 24 | 38 | 12 |
| Informing future organisation programs of work, resource development and/or resource allocation | 24 | 21 | 21 | 29 | 6 |
| Informing organisational advocacy priorities | 21 | 24 | 27 | 21 | 9 |
| Supporting public advocacy | 15 | 24 | 29 | 21 | 12 |
| Supporting direct advocacy to government decision-makers | 15 | 21 | 32 | 21 | 12 |

Figure S1: Perspectives of non-government Food-EPI Australia participants on personal impact of the initiative in March 2017 (n = 53)

Figure S2: Perspectives of non-government Food-EPI Australia participants on impact of the initiative in April - May 2020 (n = 34)

Figure S3: Reported utilisation of the Food-EPI Australia findings by non-government participants in April – May 2020 (n = 34)

Figure S4: Perspectives of non-government Food-EPI Australia participants on opportunities to strengthen the initiative in April – May 2020 (n = 34)
