# Supplementary material for: Benchmarking for accountability on obesity prevention: evaluation of the Healthy Food Environment Policy Index (Food-EPI) in Australia (2016–2020)
Source: Public Health Nutr. 2021 Oct 28;25(2):488–97. doi: 10.1017/S136898002100447X (PMC8883784; doi:10.1017/S136898002100447X)
Supplement: Supplementary file 1 [file S136898002100447Xsup.zip › S136898002100447Xsup004.docx]

**Supplementary File 2 - Food-EPI Australia evaluation surveys**

**Short-term impact survey (conducted in March 2017)**

**1. Please rate your level of agreement with the following statements relating to your involvement in the Food-EPI Australia project to date. This includes your involvement in the workshops, prioritisation process, communications about the project, interactions with other participants and release of the findings:**

| **As a result of participation in the project:** | **Strongly disagree** | **Disagree** | **Neutral** | **Agree** | **Strongly agree** |
| --- | --- | --- | --- | --- | --- |
| a) I have increased knowledge of food environments and related policy in my State/Territory |  |  |  |  |  |
| b) I have increased knowledge of food environments and related policy at the Federal level |  |  |  |  |  |
| c) I have increased knowledge of current best practice/what governments are doing globally |  |  |  |  |  |
| d) I have made new connections or strengthened existing relationships with non-government professionals |  |  |  |  |  |
| e) I have made new connections or strengthened existing relationships with people working for government |  |  |  |  |  |

**2. In what ways has the information provided in the workshops or project findings influenced your work or the work of your organisation (for example, new perspectives or ideas, changes to priorities, plans, practices, resources, structures etc.)? Please comment.**

**3. In what ways have strengthened relationships or new connections (formed as a result of this project) influenced your work or the work of your organisation (for example, sharing information, opportunities to collaborate, increased awareness of relevant organisations, individuals or projects)? Please comment.**

**4. Have you shared the project findings with other individuals within or external to your organisation? Please comment.**

**5. Please provide any additional information about your experience of participating in the Food- EPI expert panel**

**Medium-term impact survey (conducted in April – May 2020)**

**1. What type of organisation do you primarily work for?**

- University or other research institution
- Not-for-profit organisation or consumer/civil society organisation
- Health service or health network
- Other

**2. How have you personally been involved in the Food Policy Index project? (select all that apply)**

- Planning and development of the Food Policy Index for Australia
- Attending a workshop to assess government progress (conducted in 2016)
- Contributing to the development and prioritisation of priority actions (conducted in 2016)
- Read and/or shared the first Food Policy Index report (released in 2017)
- Read and/or shared the progress update to the Food Policy Index report (released in 2019)
- Other (please specify):_______________________

**3. From your perspective, what aspects of the initial 2017 Food Policy Index reports were most valuable?**

- Progress of a particular jurisdiction (e.g., the state in which you work)
- Comparisons between jurisdictions
- Priority recommendations for each government
- Examples of international best practice benchmarks
- Media coverage
- None
- Other (please specify):_______________________

Other comments or additional information about aspects of the initial 2017 Food Policy Index reports that were valuable:

|  |
| --- |

**4. From your perspective, how was the 2019 update of the Food Policy Index project (that reported progress of each jurisdiction against recommendations made in the 2017 reports) valuable, if at all?**

- Keeping governments accountable
- Maintaining awareness and discussions about the importance of healthy food environments
- Updated/additional evidence about government progress towards creating healthier food environments
- Other (please specify):_______________________
- Not valuable
- **N/A – I haven’t seen the 2019 update of the Food Policy Index project**

Other comments or additional information about the value of the 2019 update of the Food Policy Index project:

|  |
| --- |

**5. From your perspective, how much attention did the findings of the 2019 update of the Food Policy Index project receive from the your organisation or affiliated group relative to the initial 2017 results?**

- More attention
- About the same amount of attention
- Less attention
- Unsure

Other comments or additional information regarding the level of attention paid to the Food Policy Index project by your organisation of affiliated group more generally:

|  |
| --- |

6. **To what extent did you or your organisation/affiliated group utilise the findings of the Food Policy Index project (either 2017 or 2019 results) in:**

|  | **Not at all used** | **Used to a small extent** | **Used to a moderate extent** | **Used a lot** | **Unsure** |
| --- | --- | --- | --- | --- | --- |
| a) Supporting direct advocacy to government decision-makers |  |  |  |  |  |
| b) Supporting public advocacy |  |  |  |  |  |
| c) Informing organisational advocacy priorities |  |  |  |  |  |
| d) Informing future organisational programs of work, resource development and/or resource allocation |  |  |  |  |  |
| e) Increasing awareness and understanding of best practice obesity prevention policy |  |  |  |  |  |

Other comments or additional information about how the findings of the Food Policy Index project were utilised by you or within your organisation/affiliated group:

|  |
| --- |

**7. From your experience, to what extent were the findings of the Food Policy Index project (either 2017 or 2019 results) utilised throughout your professional networks:**

|  | **Not at all used** | **Used to a small extent** | **Used to a moderate extent** | **Used a lot** | **Unsure** |
| --- | --- | --- | --- | --- | --- |
| a) As part of informal discussions |  |  |  |  |  |
| b) As a discussion point at formal meetings or events |  |  |  |  |  |
| c) Sharing of the report and/or project website (e.g., via email or newsletter) |  |  |  |  |  |

Other comments or additional information about how the findings of the Food Policy Index project were utilised throughout your professional networks:

|  |
| --- |

**8. Thinking about the impact of the Food Policy Index project on yourself, please rate your level of agreement with the following statements:**

|  | **Strongly disagree** | **Disagree** | **Neither agree nor disagree** | **Agree** | **Strongly agree** |
| --- | --- | --- | --- | --- | --- |
| a) The project and its findings influenced my understanding of best practice policies to promote healthy food environments |  |  |  |  |  |
| b) The project and its findings influenced my understanding of the level of policy action among Australian governments to promote healthy food environments |  |  |  |  |  |
| c) The project and its findings influenced my understanding of what other countries are doing to promote healthy food environments |  |  |  |  |  |

Other comments or additional information about the impact of the Food Policy Index project on yourself:

|  |
| --- |

9. **Thinking about the impact of the Food Policy Index project on organisations or groups working to improve the healthiness of food environments, please rate your level of agreement with the following statements:**

|  | **Strongly disagree** | **Disagree** | **Neither agree nor disagree** | **Agree** | **Strongly agree** | **Unsure** |
| --- | --- | --- | --- | --- | --- | --- |
| a) The project and its findings encouraged **collaboration/ strengthened relationships between government officials** and non-government organisations/groups working to improve the healthiness of food environments |  |  |  |  |  |  |
| b) The project and its findings encouraged collaboration / strengthened relationships between different non-government organisations and groups working to improve the healthiness of food environments |  |  |  |  |  |  |
| c) The project and its findings increased alignment of advocacy efforts among non-government organisations and groups working to improve the healthiness of food environment |  |  |  |  |  |  |

Other comments or additional information about the impact of the Food Policy Index project on organisations or groups working to improve the healthiness of food environments:

|  |
| --- |

**10. We currently plan to repeat the full assessment of the Food Policy Index every 4 years, with a progress update every 2 years. Do you recommend changes to how often we conduct the process?**

- Yes
- No
- Unsure

If yes, what changes would you suggest to the frequency of assessments?

|  |
| --- |

**11. How could the project processes be improved next time? (select all that apply)**

- Individual rating of government policies and actions online (rather than in a workshop setting)
- Focusing stakeholder workshops on developing and prioritising actions for each government
- Independent facilitator of workshops
- Refinements to the report format (please specify):_______________________
- Greater focus on sharing the results and knowledge mobilisation
- Other (please specify type):_______________________

Other comments about how the project processes could be changed next time:

|  |
| --- |

**12. We are considering incorporating issues related to environmental sustainability of food systems as part of the Food Policy Index. How important do you think it is to incorporate environmental sustainability as part of this initiative?**

- Very important
- Moderately important
- Slightly important
- Not important at all
- Unsure

**How do you think the incorporation of environmental sustainability might be received by senior government policy makers?**

|  |
| --- |

**13. Do you have any other comments about the Food Policy Index project?**

|  |
| --- |
